# Human Motion Understanding for Selecting Action Timing in Collaborative Human-Robot Interaction

**DOI:** 10.3389/frobt.2019.00058

**Published:** 2019-07-16

**Authors:** Francesco Rea, Alessia Vignolo, Alessandra Sciutti, Nicoletta Noceti

**Affiliations:** ^1^Robotics Brain and Cognitive Sciences (RBCS), Istituto Italiano di Tecnologia, Genova, Italy; ^2^CONTACT, Istituto Italiano di Tecnologia, Genova, Italy; ^3^Dipartimento di Informatica, Biongegneria, Robotica e Ingegneria dei Sistemi (DIBRIS), Università degli Studi di Genova, Genova, Italy

**Keywords:** human motion understanding, action synchronization, motion signature, optical flow, human-robot interaction, view-invariance

## Abstract

In the industry of the future, so as in healthcare and at home, robots will be a familiar presence. Since they will be working closely with human operators not always properly trained for human-machine interaction tasks, robots will need the ability of automatically adapting to changes in the task to be performed or to cope with variations in how the human partner completes the task. The goal of this work is to make a further step toward endowing robot with such capability. To this purpose, we focus on the identification of relevant time instants in an observed action, called *dynamic instants*, informative on the partner's movement timing, and marking instants where an action starts or ends, or changes to another action. The time instants are temporal locations where the motion can be ideally segmented, providing a set of primitives that can be used to build a temporal signature of the action and finally support the understanding of the dynamics and coordination in time. We validate our approach in two contexts, considering first a situation in which the human partner can perform multiple different activities, and then moving to settings where an action is already recognized and shows a certain degree of periodicity. In the two contexts we address different challenges. In the first one, working in batch on a dataset collecting videos of a variety of cooking activities, we investigate whether the action signature we compute could facilitate the understanding of which type of action is occurring in front of the observer, with tolerance to viewpoint changes. In the second context, we evaluate online on the robot iCub the capability of the action signature in providing hints to establish an actual temporal coordination during the interaction with human participants. In both cases, we show promising results that speak in favor of the potentiality of our approach.

## 1. Introduction

Working efficiently together relies on mutual understanding of the two agents, often based on intuitive and fast comprehension of what the partner is doing and when it is the right moment to act. Humans are quite effective in establishing this type of coordination with their colleagues (Flanagan and Johansson, 2003), also thanks to a set of pro-social abilities naturally developed since childhood (see e.g., Vignolo et al., 2017). In view of the adoption of novel technologies in the future workplaces and industrial environments, it becomes crucial to understand how to endow also these new artificial “interactors” with similar skills. The goal is to make the transition from human-human interaction to human-technology interaction smoother, easier and not too cumbersome for the human workers (Sciutti et al., 2018).

The presence of robots is envisaged in our future industries. Importantly, unlike traditional industrial robots, whose actions are precisely programmed and scheduled *a priori*, future robots are expected to perform more unconstrained activities, being able to adapt to frequent changes in the specific task to be handled and to the collaboration with users with limited technical expertise in the use of the machine. In these contexts, the robot will need to autonomously understand—to a certain degree—the activity^1^ it is confronted with and—additionally—to be able to select the appropriate timing for its actions, ideally by adapting to its human partner.

In this paper we take a step toward endowing robots with action understanding and synchronization abilities, by focusing on the identification of relevant time instants in an observed action, informative of the partner's movement timing. The instants can be interpreted as temporal locations where the time signal describing a motion can be ideally segmented, providing a set of primitives that can be used to build a temporal signature of the action and finally support the understanding of the dynamics and coordination in time.

We propose to identify such relevant instants by exploiting motion information embedded in the so-called *dynamic instants*, i.e., time instants in which the dynamic of an action is subject to a change, that may be due to variations in velocity, acceleration, or direction of motion. The original formulation of the dynamic instants date back to the 80's (Rubin and Richards, 1985; Gould and Shah, 1989) and has been later renewed by Rao et al. (2002) to mathematically prove that they are preserved after projective transformations, and as a consequence, that their detection is robust to viewpoint changes. Other methodological approaches detect successfully dynamic instants (Dollar et al., 2005; Buchsbaum et al., 2011; Gong et al., 2014) and prove them to be meaningful tools for coordination (Chou et al., 2018), but our method adopts a different computational approach and detects dynamic instants without either a priori knowledge or relevant amount of training data.

**Figure 1:**
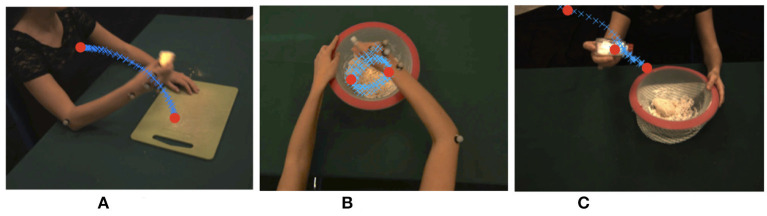
Examples of dynamic instants detected for a variety of actions (as eating, mixing, and sprinkling the salt) and viewpoints. Dynamic instants are marked in red circles, while blue crosses show the dynamic evolution of the action over time. **(A)** Eating, **(B)** Mixing, **(C)** Spreading salt.

In our approach, dynamic instants are identified as minima of the velocity profile, which we directly derive from the optical flow, obtaining a compact yet highly informative measure representing the motion evolution over time (Noceti et al., 2015). The dynamic instants we obtain in this way are classified as instants where an action is starting, ending, or changing (Noceti et al., 2018). In other words they are distinguishable events during continuous movements which could help segmenting the action and predicting the timing of a repetitive motion (see in Figure 1, in red, examples of dynamic instants detected for three different actions). Notably we look for instants that could be robust across different views and without requiring any *a priori* information about the actor's shape or position. As a consequence, we are able to process sequences of motion observed at different levels of granularity (e.g., performed with the entire arm or with a single hand) guaranteeing the capability of adapting to a variety of scenarios.

While in our previous work (Noceti et al., 2018) we experimentally assessed the accuracy in the detection of our dynamic instants, in this paper we also validate the use of a string-based description of the partner's motion to support human-robot collaboration in two contexts. First, we show that such a description could facilitate the understanding of which type of action is occurring in front of the robot, when the activities that can be performed by the human partner can be of a various nature. Second, we verify that it can help in establishing an actual temporal coordination online with naive users, once the activity has been determined and shows a certain degree of periodicity. In both contexts, action representation is approached by segmenting the observed complex action into intervals, delimited by pairs of temporally adjacent dynamic instants and composed by the values in the velocity profile occurring between the two.

To address *action understanding* in free interaction, we derive from the sequence a motion signature based on the use of strings encapsulating the sequence of dynamic instants and intervals over time. Next, the signatures are compared to explore similarities with known models using a string kernel (Noceti and Odone, 2012), which is able to capture similarities in terms of sub-strings, i.e., in this contexts sub-movements shared by the actions.

To validate this approach we focus on the batch analysis of a dataset we collected in-house depicting a variety of kitchen activities observed from multiple points of view. In the context of Computer Vision, interesting results based on action classifiers have been achieved (Chou et al., 2018). Our approach may be related to works on action recognition, as Rao et al. (2002), Shi et al. (2008), Shao et al. (2012), and Cabrera and Wachs (2017), but differently from those works, we are not pursuing a recognition task, rather we explore the feasibility of using such signature to address motion understanding from a more general perspective, i.e, to investigate (i) What kind of information is embedded in the representation, and (ii) How far a relatively poor description, as the one we propose, can bring the system in the task of understanding actions properties.

In the second application context, *action synchronization* is based on the assumption that the activity to be monitored and executed together is summarily known in advance and has a certain degree of periodicity. This task however poses a different set of challenges: it requires being able to extract action timing in real time and a process to allow for adaptation of the robot action. Indeed, although the average execution time of the action can be known *a priori*, significant differences might exist among different operators and slight variations in the task execution—as simple as changing the relative positions of the tools or objects to be used or factors like training or tiredness—can lead to significant changes in human timing. To test this skill we propose an interactive scenario recalling a collaborative interaction in a table-top work environment with the humanoid robot iCub and we validate the interaction with human participants, based on an online version of the algorithm.

We experimentally show that the proposed method detects rather accurately the dynamic instants of the variety of actions included in the dataset, across the different views presented. Moreover, the action segmentation and reconstruction approach based on dynamic instants allows up to a certain extent to categorize the variety of activities recorded. When tested in the synchronization task, the system shows the feasibility of its use in online settings, enabling the reliable detection of dynamic instants. For actions where each instance can be delimited by pair of dynamic instants—i.e., actions composed by a single primitive—this process also allows the estimation of action duration. This is the feature that we exploit to allow for synchronization in human-robot collaboration for rhythmic activity and we demonstrate the robustness of the method even in presence of relevant modifications in action (e.g., in the rhythm or the direction) execution within and between users.

The remainder of the paper is organized as follows. Section 2, which introduces our approach to build the motion descriptor, is followed by section 3 where we assess the method in an offline scenario. Section 4 considers the application to enable adaptive synchronization in HRI, that we experimentally evaluate considering the methodologies in section 5. The results of the online experimental analysis are reported in section 6, while section 7 is left to a final discussion.

## 2. Motion Signature From Videos

In this section we describe our approach to build the motion description, which is based on deriving a motion signature composed as the sequence of dynamic instants and intervals over time. In the following we review each step in our video-based analysis, to finally describe the motion signature adopted. To summarize, we envisioned the adoption of a compact motion description based on strings, as a way to compactly describe all the kinematics features relevant to the task, to facilitate the comparison and similarity assessment of a variety of different actions.

### 2.1. Extracting Motion Features

**Figure 2:**
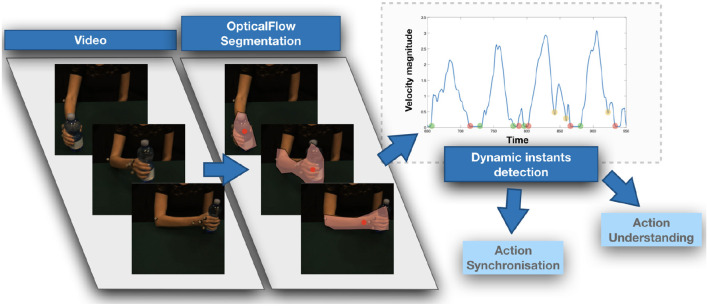
A visual representation of the computation of low-level motion features. Optical flow is computed and thresholded to detect the moving region, where the information is collapsed in a centroid associated with the average velocity magnitude. Such value, analyzed of time, is the signal on which dynamic instants are detected.

Given a video stream, we start by applying at each frame the method (Vignolo et al., 2016) to identify and describe a moving region. The method relies on the computation of the optical flow using Farnebäck (2003) to provide an estimate of the apparent motion vector in each pixel (see Figure 2, left). Next, a motion-based image segmentation is applied to detect the region of interest. The segmentation leverages a thresholding of the optical flow magnitude to identify points with significant motion information. To such points, a standard approach for perceptual grouping is applied to identify the connected components: given a binary image, obtained after thresholding, we consider eight-connected pixels as belonging to the same object. Finally the largest component is selected as a representation of the structure of interest moving in the scene. We will refer to it as R(t), i.e., the moving region of time *t*. Given a video V of length *T*, we finally obtain a sequence of moving regions, i.e., with V we associate a sequence ${\left[R(t)\right]}_{t=1}^{T}$.

**Figure 3:**
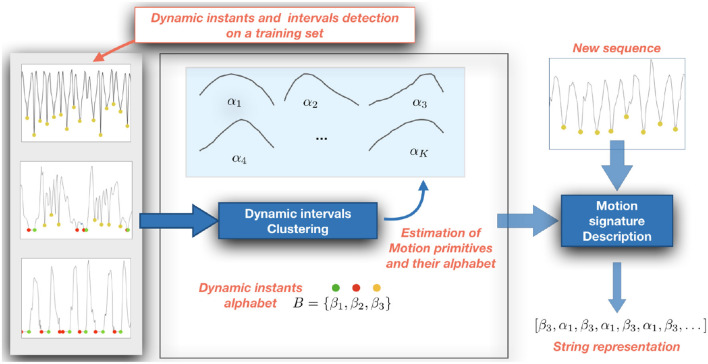
A visual representation of the computation of our motion signature.

Let **u**_*i*_(*t*) = (*u*_*i*_(*t*), *v*_*i*_(*t*)) be the optical flow components associated with point ${\text{p}}_{i}\left(t\right)\in R\left(t\right)$, and N the size of the region, i.e., the number of pixels in it.

The motion of the region can be compactly yet effectively described by computing the average of the optical components: $V\left(t\right)=\frac{1}{N}\underset{{\text{p}}_{i}\left(t\right)\in R\left(t\right)}{\sum }||{\text{u}}_{i}\left(t\right)||$. By combining the obtained representations over time, we finally compose a temporal description of the occurring dynamic event: from the video V we derive the sequence ${\left[V(t)\right]}_{t=1}^{T}$ (see an example in Figure 2, right).

### 2.2. Detecting Dynamic Instants and Intervals

As in Noceti et al. (2018), we derive the dynamic instants as the local minima of the velocity profile describing the apparent motion in a sequence. From a more formal point of view, dynamic instants are locations $\hat{t}$ in time in the interval [1…*T*] such that $V^{\prime }\left(\hat{t}\right)=0$ and $V^{\prime \prime }\left(\hat{t}\right)>0$. Such time locations can be categorized as START, STOP, and CHANGE points. START points are those instants in which the motion starts after a rest, while STOP points correspond to the end of an action followed by an interval in which no motion is occurring. CHANGE points refer to time instants between two atomic action units, performed continuously in time. As for the detection procedure, given the locations $\hat{t}$ of the local minima in the velocity profile, we analyse each of these points to assign them a label. More specifically, if the velocity of a certain dynamic instant $\hat{t}$ is above a threshold τ, then it is assigned the CHANGE label. Conversely, if the velocity is very close to zero, meaning lower than a threshold ϵ referring to the floating point precision of the machine, then we verify if a plateau is present after the current location, in which the velocity persists in this status. To this purpose, we simply count how many consecutive values of the velocity verify the above condition, and detect a plateau if they are at least 5 (value chosen empirically). If this is the case, then we assign the labels STOP and START to, respectively, current and next dynamic instant, otherwise a CHANGE is again detected.

At this point, we can easily represent our original video V as a set

(1) $$\begin{array}{r} M=\left\{\Gamma ,\Sigma \right\} \end{array}$$

where $\Gamma =\left\{{\hat{t}}_{1},{\hat{t}}_{2},\ldots ,{\hat{t}}_{D-1},{\hat{t}}_{D}\right\}$ is the set of D dynamic instants locations in time, i.e., the ordered sequence of the time instants in the temporal reference system of the video V, while $\Sigma =\left\{V\left({\hat{t}}_{1}:{\hat{t}}_{2}\right),\ldots ,V\left({\hat{t}}_{D-1}:{\hat{t}}_{D}\right)\right\}$ is the corresponding set of dynamic intervals (the notation ${\hat{t}}_{k}:{\hat{t}}_{k+1}$ refers to all the values in the velocity vector between the two dynamic instants).

The informative content embedded in *M* is exploited to address the action synchronization task, while for the action understanding in more unconstrained settings a further step is needed, which is described in the following section.

### 2.3. Building a Motion Signature

We obtain a motion signature on the action sequence deriving a string representation from the set *M*. To this purpose, we first collect a training set of dynamic intervals which we cluster using K-Means (Duda et al., 2012) to obtain a set of K prototypical dynamic intervals profiles (i.e., the centroids of the clusters). To allow for the clustering, we first downsample each dynamic interval to a fixed length, set to 8 in our experiments.

Each prototype is associated with a character label, that compose the alphabet *A* = {α_1_, …, α_*K*_}. Then, given a sequence described according to Equation (1), we compute the distance between each dynamic interval and the prototypes, and associate the interval with the character in *A* corresponding to the closest one. This leads us to map a sequence of temporally ordered dynamic intervals into a string *s*_1_. As a distance measure, we employ the Euclidean distance between the dynamic interval and the cluster centroids.

We further enrich the string *s*_1_ by combining it with another string *s*_2_ derived from the sequence of dynamic instants. To this purpose, we define a second alphabet *B* = {β_1_, β_2_, β_3_} in which the characters refer to the classes of, respectively, START, STOP, and CHANGE points, and associate each dynamic instant detected in an action sequence with the corresponding label.

The combination of the two strings *s*_1_ and *s*_2_ in a single one, by putting the characters in an ordered sequence reflecting their original temporal occurrence in the video, provides the final string *s* representing our motion signature of the sequence.

## 3. Assessing Dynamic Instants

In this section we validate our strategy in an offline setting, considering the *Multimodal Cooking Actions* (MoCA) dataset^2^, we acquired in-house, that includes videos depicting cooking activities observed by three different view points. The goal of this experimental assessment is to evaluate the quality of our motion signature in a simple motion understanding task, we implement in the form of a matching problem between actions. With this analysis, we want to highlight how powerful is the relatively poor description we adopt, speaking in favor of its inherent high level of expressiveness. In the reminder, we first briefly describe the MoCA dataset, and then discuss the experimental analysis in details.

### 3.1. Multimodal Cooking Actions Dataset

The *Multimodal Cooking Actions* (MoCA) dataset is a multimodal dataset including MoCap data and video sequences acquired from multiple views of upper body actions in a cooking scenario. Differently from available datasets acquired in similar environments (see e.g., Damen et al., 2018) it has been collected with the specific purpose of investigating view-invariant action properties in both biological and artificial systems, and in this sense it may be of interest for multiple research communities in the cognitive and computational domains.

The dataset includes 20 cooking actions, namely *grating a carrot, cutting the bread, cleaning a dish, eating, beating the eggs, squeezing the lemon, using the mezzaluna knife, stirring a mixture, opening a bottle, using a pan, crushing the garlic, peeling a potato, pouring water, reaching an object, rolling the dough, mixing a salad, sprinkling salt, spreading the cheese, cleaning the table, transporting an object*. The actions are performed by a single user acting in a common environment which is observed by a set of three cameras acquiring the video sequences synchronously (sample frames from the three views are reported in Figure 1): a lateral view, a viewpoint slightly above the subject's head (reminiscent of an ego-centric point of view), and a frontal view. As it can be noticed, the motion appearance can be significantly influenced by the viewpoint, which impacts not only on the direction but also on the magnitude of the average optical flow vectors. For this reason, the dataset is an ideal test-bed for evaluating the tolerance to viewpoint changes of our motion representation. To this purpose, we manually annotated the dataset marking the temporal locations of START, STOP, and CHANGE dynamic instants. Notice that actions may contain only some of the dynamic instants type, e.g., repetitive actions as *mixing a salad* only contains CHANGE points. Only 17 actions out of the 20 (we left out *grating a carrot, using a pan, spreading the cheese*) allowed for an unambiguous annotation.

Most of the observed actions also involve the manipulation of objects. Each video consists in the repetition of 20 instances of the atomic action. For each action a pair of videos has been acquired, so to have available a training and a test sequence. Cameras acquire images of size 1, 293 × 964 at a rate of 30*fps*.

### 3.2. Experimental Analysis

We start by evaluating the accuracy of our method in detecting the dynamic instants, moving then to a preliminary analysis on the application to action recognition. If not otherwise stated, in all the experiments reported in the paper, the threshold applied to the optical flow estimates for the identification of the moving region has been set to 2, while the value of τ (see section 2.2) has been fixed to 5. The values has been experimentally selected on the training set.

#### 3.2.1. Dynamic Instants Detection

**Figure 4:**
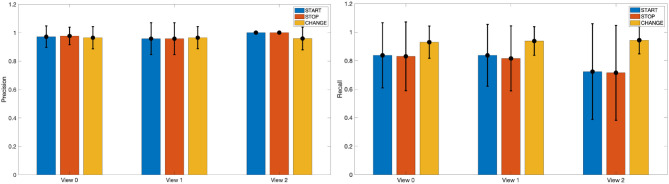
A visual impression of *Precision* and *Recall* values for the detection of START, STOP, and CHANGE dynamic instants in the different views of the MoCA dataset.

In Figure 4 we report a visual impression on the performance of the dynamic instants detection in terms of *Precision* and *Recall* values obtained on the different views of the MoCA dataset. A dynamic instant with label *L* is successfully detected at time *t*, if it exists in the ground truth an annotated instant at time *t*′ with same label and such that |*t* − *t*′| ≤ Δ*T*. In the experiments, we fixed Δ*T* = 6, corresponding to $\frac{1}{5}s$.

On the left panel of Figure 4 we show with bars the precision, averaged across the different actions on the test set, for each type of dynamic instants (START, STOP, CHANGE) which have been grouped with respect to the view. The lines on top of the bars represent the standard deviations. It can be noticed how the *Precision* values are all very high, and stable across views and class of dynamic instants. On the right panel of the same figure, we report instead the corresponding *Recall* values, from which we can observe a higher robustness (i.e., higher *Recall*) in the detection of CHANGE instants in comparison to the detection of START and STOP points, that are nevertheless characterized by *Recall*'s very close to 0.80.

A closer look to the performance associated with each action shows uneven results. To highlight this aspect we report in Table 1 the values of *Precision* and *Recall* we obtained for each action. In this analysis, we consider as a reference the data from View 2, which appears to be the weakest in terms of detection results. For completeness we also included in the table the number of true positive, false negative, and false positive detections. The latter, in particular, can occur also in actions not including them (and in such case their influence can not be evaluated with the *Recall* because the number of true positives would be null). It is easy to note that some actions are particularly affected by the problem of missed detections. An example is *Cutting the bread* for which, the pause the actor makes between consecutive actions is too short to be successfully detected. A similar example is the action *Sprinkling salt*, which in addition is also affected by the presence of many false positives, most likely due to the high speed of the action; *Pouring the water* is also characterized by a small amount of false positives, that in this case are however due to the apparent motion of the water inside the container manipulated by the subject. We finally observe that actions characterized by a limited variation in space, as *opening a bottle* and *squeezing a lemon*, can be affected by missed detection, mainly due to the limited amount of information derived from the motion segmentation stage.

**Table 1:** A detailed analysis of *Precision, Recall, True Positives, False Positives*, and *False Negatives* obtained with our method on the View 2 of the MoCA dataset.

		**START**					**STOP**					**CHANGE**				
	**Length**	**Prec**.	**Rec**.	**TP**	**FP**	**FN**	**Prec**.	**Rec**.	**TP**	**FP**	**FN**	**Prec**.	**Rec**.	**TP**	**FP**	**FN**
Cutting	1,717	1	0.25	4	0	12	1	0.25	4	0	12	0.84	1	107	20	0
Cleaning	388	–	–	–	0	–	–	–	–	0	–	1	1	41	0	0
Eating	1,756	1	0.93	26	0	2	1	0.89	25	0	3	–	–	–	3	–
Eggs	615	–	–	–	1	–	–	–	–	1	–	1	0.82	56	0	12
Lemon	580	–	–	–	3	–	–	–	–	3	–	1	0.76	32	0	10
Mezzaluna	543	–	–	–	0	–	–	–	–	0	–	0.98	1	54	1	0
Mixing	456	–	–	–	0	–	–	–	–	0	–	1	1	38	0	0
Openbottle	2,070	1	0.72	26	0	10	1	0.69	25	0	11	1	0.78	56	0	16
Pestling	455	–	–	–	0	–	–	–	–	0	–	0.95	1	21	1	0
Pouring	1,050	1	0.90	36	0	4	1	0.90	36	0	4	–	–	–	7	–
Reaching	1,900	1	1	38	0	0	1	1	38	0	0	–	–	–	0	–
Rolling	755	–	–	–	0	–	–	–	–	0	–	1	0.98	43	0	1
Salad	720	–	–	–	0	–	–	–	–	0	–	1	0.98	39	0	1
Salt	1,180	1	0.24	5	0	16	1	0.24	5	0	16	0.75	1	63	21	0
Table	500	–	–	–	0	–	–	–	–	0	–	0.98	1	43	1	0
Transporting	2,060	1	1	38	0	0	1	1	38	0	0	–	–	–	0	–

*The second column also reports the length of the sequences in frames*.

#### 3.2.2. Motion Understanding With Strings

**Figure 5:**
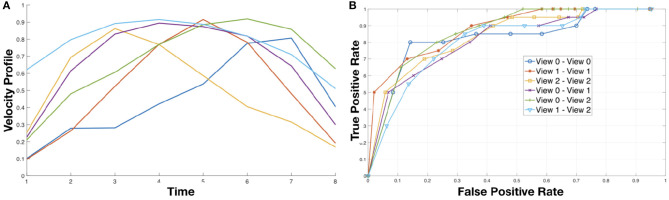
**(A)** (Left) Motion primitives obtained using K-Means to cluster the motion intervals detected between two dynamic instants. **(B)** (Right) The ROC curves of simple classifier based on thresholding the similarity measures between actions across views.

To address the motion understanding task we built a dictionary of 6 primitives using all the measures extracted from the training sequences of the cooking dataset. The primitives, shown on the left in Figure 5, are characterized by a bell shaped velocity profile with different relative timing between acceleration and deceleration phases, a typical trait of human motion (Atkeson and Hollerbach, 1985; Sciutti et al., 2012).

To evaluate the expressiveness of the primitives, we set up a simple matching tasks based on a thresholding of the similarity between pairs of motion signatures. More specifically, we estimated as described in section 2.3 the motion signatures for each video in training and test set. Then, we compare each test signature with all the training signature and threshold their similarity with respect to a value δ, i.e., a pair of signatures is considered a match if their similarity is above δ. The similarity between signatures (that are strings) is done using a string kernel, and in particular the *P*-Spectrum kernel (Noceti and Odone, 2012). It evaluates the similarity between two strings counting how many substrings of length *P* the two strings have in common. Given the nature of our strings, which are in general sequences of dynamic intervals delimited by a pair of dynamic instants, in our task a reasonable value for *P* is 3. By varying the value of δ we built the ROC curve reported on the right of Figure 5. Notice that the performance of multi-view matching (View i vs. View j, with i ≠ j) is very much in line with the results obtained comparing actions in the same view. This is an evidence of the fact the motion signature embeds invariant properties of the actions.

**Figure 6:**
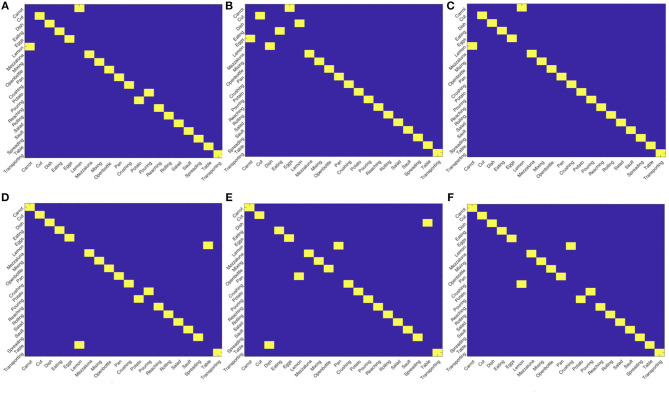
Results of the assignments between views using the Hungarian algorithm. Although not perfect, the results show a strong capability of the descriptor in highlighting affinity between actions across views. **(A)** View 0 - View 0, **(B)** View 0 - View 0, **(C)** View 0 - View 0, **(D)** View 0 - View 0, **(E)** View 0 - View 0, **(F)** View 0 - View 0.

As a further evidence of this point, we notice that the fact the matching between actions, either intra- or inter- views, does not provide perfect scores suggests the presence of groups of actions sharing some similarities. However, if we apply the Hungarian algorithm (Kuhn, 1955) to assignment problems on the similarity matrices obtained comparing the views, we obtain the results in Figure 6. Although not perfect, the quality of the obtained assignments is very promising, especially considering we start form a simple description purely based on motion. This will give us hints on possible future development, we will highlight in the final discussion, to exploit this strategy for enabling a proficient collaboration also in this challenging setting.

## 4. Using Dynamic Instants for HRI

**Figure 7:**
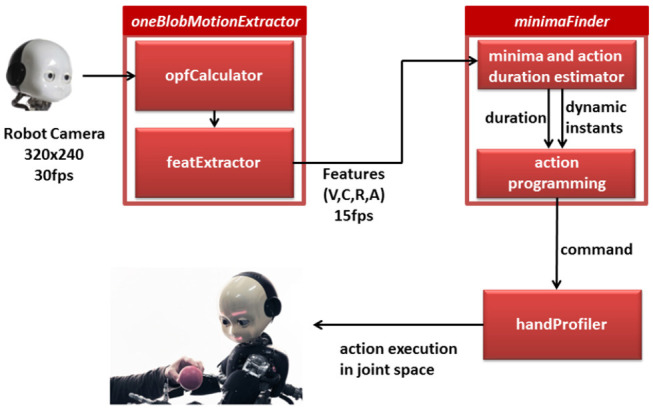
A visual sketch of the online procedure to detect dynamic instants on the and plan the consequent action of the robot.

In the previous sections we have shown that it is possible to extract dynamic instants from an offline analysis of videos of complex actions and that this information could be used to segment and describe different activities in a compact way. The possibility to detect dynamic instants online however would provide a robot with a precious information supporting action timing coordination in joint tasks. In this section we introduce the software solution that detects the dynamic instants through vision (see a visual sketch of the procedure in Figure 7). We verified the implementation with the humanoid robot iCub. To this purpose, we designed a version of the Dynamic Instant Detection algorithm that could work on-line, i.e., during the interaction and we integrated in the existing iCub software framework, based on the middleware Yarp (Metta et al., 2010). More specifically, the online dynamic instants detection follows the same process described in section 2, but leverages a sliding window of varying dimension in which the detection is performed. Assuming that the observed action is cyclic, the instant detection reliably predicts the occurrence of the successive dynamic instant. Further, the interval between successive dynamic instants represents an estimation of the action duration which in turn can be used to generate a robotic action with the appropriate duration. We can obtain reliable action coordination between the human and robot when associating the next starting event predicted for the robot action and the proper action duration. In the following we give details on each module developed to provide such coordination.

### 4.1. Online Dynamic Instants Detection in the iCub Framework

The input for the dynamic instant detection is a sequence of images from the right camera of the robot iCub, acquired with a frame rate of 15fps at the image resolution of 320 × 240. The method uses the image analysis of the software module proposed in Vignolo et al. (2016) and Vignolo et al. (2017). Thanks to this the humanoid robot iCub recognizes the presence of motion in the surrounding and segments motion that is produced by a human, i.e., by a potential interacting agent. The dynamic instants are detected from the video stream generated by the human movement on to the image plane and fed into the core software module called *oneBlobMotionExtractor*. The robot head orientation is fixed to simplify the estimation of humans motion feature. Consequently the module extracts motion features from the optic flow associated with the observed human action. The relevant features are sent to the input port of the *minimaFinder* module (see Figure 7), which analyzes in particular one feature, i.e., the velocity over time. From the analysis of the instant temporal location of the minima in the velocity profiles, the system computes the expected occurrence of future dynamic instants (see description of minimaFinder module below) and the duration of the human action. Thanks to these two key aspects of the movement, the robot plans the action indicating when the action starts (i.e., action trigger event) and its duration. The action trigger event and duration of the robot action are sent to the *handProfiler* module, which is devoted to the synthesis of biological-plausible robot actions. In the remainder of the section we provide a more detailed description of each module.

#### 4.1.1. oneBlobMotionExtractor

The analysis of the optic flow relies on two separate processes, i.e., the two classes of concurrent computing modules *opfCalculator* and *featExtractor*. The two processes address, respectively the segmentation based on motion of the moving element (i.e., blob) and the description of the blob motion through a series of features. The separation in blobs guarantees that the computation of the module leverages multi-threading and implements efficient parallel computing. In particular, the *opfCalculator* module outputs separate maps for the horizontal and vertical components of the optical flow (see section 2.1). From the computed optic flow, the *featExtractor* process analyzes the largest and most persistent moving blob. Our assumption is that only one region of interest moves in the scene. This assumption is acceptable in several human-robot interaction settings, e.g., in table top scenarios where the task of the human partner is in general of moving or passing one object or tool with his or her hand. The process provides to the rest of the Yarp network, and thus to the other modules in the software infrastructure the velocity feature and the corresponding timestamps.

#### 4.1.2. Minimafinder

Following the consideration in section 2, the module interprets the velocity profile to detect the dynamic instants in the observed actions. The module uses exclusively the velocity profile extracted by the *oneBlobMotionExtractor*. The *minimaFinder* instantiates two parallel and asynchronous processes aiming, respectively at *monitoring* and *commanding execution*. For the *monitoring* task, the *minimaFinder* reads the vector of features provided by the *featExtractor*, and it finds the minima in the dynamic progression of velocity. The values are filtered in time using a running average formulation:

(2) $$\begin{array}{r} Vel_{k}^{\;f}=\alpha \cdot Vel_{k}^{\;m}+\left(1-\alpha \right)\cdot Vel_{k-1}^{\;f} \end{array}$$

where α is a weight controlling the importance of the two terms, $Vel_{k}^{\;f}$ is the filtered velocity and $Vel_{k}^{m}$ is the measured velocity. This step helps us to eliminate small fluctuations due to noise in the sensing.

For the entire non-overlapping sliding time window, the module stores in a buffer the instant measures of velocity passed by *featExtractor* and the corresponding timestamp. Within the buffer of all the velocity measures in the time window, the *minimaFinder* extracts the global minimum.

By subtracting the timestamp of such minimum with the previous one, we estimate the temporal interval between two consecutive dynamic instants in the motion as Δ*t*_*k*_ = *t*_*k*_ − *t*_*k* − 1_ where *t*_*k*_ is the timestamp of the current minimum and *t*_*k*−1_ of the previous minimum in the velocity profile.

The temporal duration of the non-overlapping sliding time window is adjusted with the estimated human action duration Δ*t*_*k*_. The duration is filtered in order to guarantee at the same time prompt and smooth adaptation to changes in the human action pace.

In particular, assuming that the observed action is cyclic and repeatable over time, the module computes the duration using again a running average:

(3) $$\begin{array}{r} {\bar{\Delta t}}_{k}=\beta \cdot \Delta t_{k}+\left(1-\beta \right)\cdot {\bar{\Delta t}}_{k-1} \end{array}$$

with β balancing the importance of the two terms, and ${\bar{\Delta t}}_{k}$ representing the estimated time interval before the next dynamic instant.

The occurrence of the next dynamic instant, hereafter also called *targetTimestamp*, ${\hat{t}}_{k+1}$, is then obtained by summing to estimated interval the last detected dynamic instant ${\hat{t}}_{k+1}\;=t_{k}\;+\;{\bar{\Delta t}}_{k}$.

In the *command execution* phase, when the internal clock is close to the *targetTimestamp*, i.e., in the interval ${\hat{t}}_{k+1}\pm \epsilon$, the *minimaFinder* sends to the controller of the whole body motion of iCub the command $\text{E}XE\text{C}\left({\hat{t}}_{k+1},{\bar{\Delta t}}_{k}\right)$. The first parameter sets the timing of the robot action in correspondence to the (predicted) dynamic instant, the second parameter controls the duration of the next action to a value corresponding to the estimated time interval.

**Figure 8:**
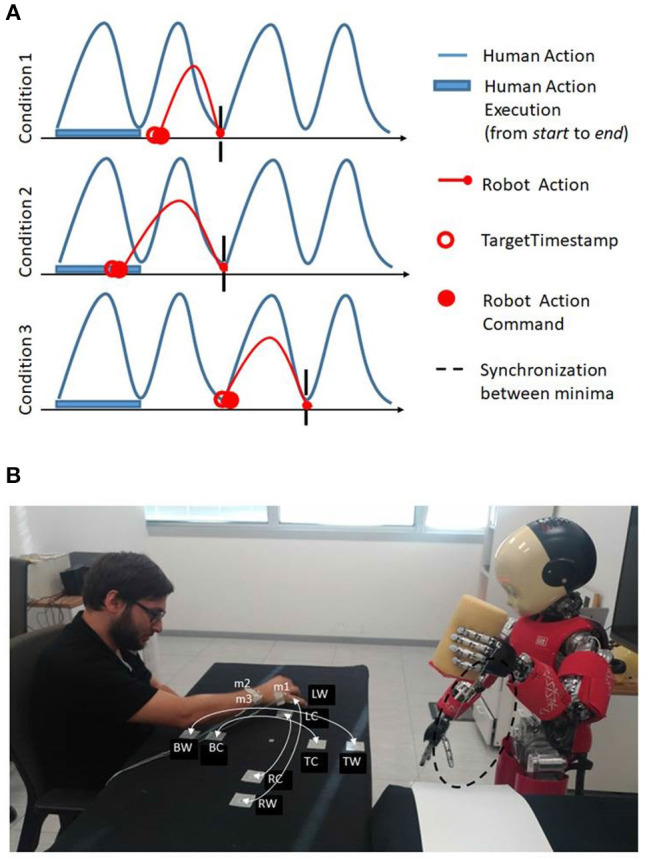
**(A)** On the top, the three strategies adopted by minimaFinder in relation to three possible conditions: (1) The new *targetTimestamp* occurs after the end of the action execution time; (2) The new *targetTimestamp* occurs before the end of the previous action; (3) The new action execution occurs after the end of the successive action execution. **(B)** On the bottom, experimental setup where the robot iCub stands in front of the subject observing his or her movements. The subject performs reaching movements between the BW-TW, BC-TC, RW-LW, RC-LC where B: bottom, T: top, R: right, L: left, C: central, and W: wide. The optotrack active markers are indicated with m1, m2, m3 labels. The iCub robot performs stamping action whose trajectory is highlighted in dashed black line.

In the first condition the *minimaFinder* waits until the *targetTimestamp* is reached to send the new command. In the second condition the *minimaFinder* immediately executes another action command but the action duration is computed as ${\bar{\Delta t}}_{k}-\epsilon$, it is shortened to guarantee next synchronization between robot and human minima. In the third condition the *minimaFinder* immediately executes another action with same duration as the temporal interval between dynamic instants. The *minimaFinder* avoids to send new commands to the controller if the controller has not terminated the action. At the end of the action execution the *minimaFinder* adopts three strategies in relation to three possible conditions (Figure 8, on the top: [1] The new *targetTimestamp* occurs after the end of the action execution time; [2] The new *targetTimestamp* occurs before the end of the previous action execution time of ε seconds; [3] The new action execution occurs after the end of the next time window).

#### 4.1.3. Handprofiler

The *handProfiler* module generates and executes wholebody biologically plausible movements, given a desired trajectory and the desired action duration. The only constraint is that the trajectory of each motion (or part of motion) can be represented as a portion of an ellipse. Ellipses have been chosen for their characteristic of generic curves. Thanks to their parametric definition, ellipses reproduce a great number of trajectories with different curvatures, ranging from circles to quasi-straight curves. The robot executes the 3D motion guaranteeing at the same time that the velocity of the end-effector (center of the palm in the iCub robot) visits the different sections of the ellipse respecting a law of biological motion, and more specifically the *Two-Third Power Law* (Richardson and Flash, 2002; Noceti et al., 2017). The selection of biologically plausible motion for the humanoid robot is motivated by findings that suggest that this choice promotes improved coordination in HRI collaborative tasks (e.g., Bisio et al., 2014). Once generated, the position in time of every step action are saved as trajectory in the space of joint angles. To perform such conversion the module computes the inverse kinematic in realtime during the action execution. This establishes perfect repeatability in the trajectory execution and fast generation of the action, since inverse kinematic is only computed during the first execution. The user can change the duration of the action. In such case, the module automatically recomputes the temporal scale factor that changes proportionally all the time intervals between consecutive joint positions in file. Since the procedure does not change the shape of the velocity profile, it also maintains the action compatible with the Two-Third Power Law.

## 5. Experimental Methods

In order to assess the ability of the robot to coordinate its actions with a human partner by using the online dynamic instant detection described in the previous section, we considered a scenario recalling a collaborative interaction in a table-top work environment. We emulate a situation in which the human partner is performing a repetitive task at his or her own pace and the robot has to perform a subsequent action, adapting to the partner's timing. We explicitly want to assess the ability of the robot to understand the appropriate action timing with no exact specification of the actions performed by the human partner, in terms of trajectories or movement speed. The assumption however is that the human action will be cyclic. In the experiment the participants have to move a stamp on a table toward different targets at their natural rhythm, mimicking a stamping task. The robot, which is in front of the human partner, with the cameras looking at his movements, has to perform a stamping action itself orthogonal to the human partner's trajectories on a different target, in coordination with him (see the experimental setup in Figure 8, on the bottom).

We tested the system to observe how the robot adapts to different people by recording 7 naive subjects (5 males and 2 females, average age 29 ± 10 years old). More in detail, the participants have been instructed to move the stamp on different locations marked on a table, first 15 times from point BW to the point TW and back (phase w-v, wide, and vertical), then the same for LW and RW (phase w-h, wide and horizontal), BC and TC (phase s-v, small and vertical), LC and RC (phase s-h, small and horizontal) (see Figure 8, on the bottom). The target locations have been indicated on the table with small circles and participants have been instructed to stamp precisely in the circle. To maintain the attention of the participants to the task, the stamp was filled with water and they were asked to be careful not to pour it during the transportation of the object. Moreover, we asked the subjects to be focused on their own motion and not consider the robot moving in front of them in order to avoid motor contagion and make them maintain their natural speed (Vannucci et al., 2017). This choice was made to guarantee that the adaptation measured during the experiment was a result of the robot's ability to achieve synchronization and not a consequence of participants' tendency to adapt to the partner.

We collected kinematic data of each participant and the robot through an Optotrak motion capture system (NDI) with three active infrared markers placed on the right hand of the participant (two on the left and right part of the wrist and one on the first knuckle of the little finger, in order to be sure to have always at least one marker well visible from the Optotrak) and 1 marker on the left hand of the robot (with 100 Hz as frequency of recording). We also collected the motion features extracted from the optical flow of the observed action by the module oneBlobMotionExtractor, in order to compare in post-processing the ground truth of the human speed (recorded with the Optotrak system) and the speed measurement computed by the robot from the camera images online. The camera is static and monitors human movements to simplify the task, however the eventual egomotion can be compensated by existing iCub software.

In the experiment, the robot detects the dynamic instants in the observed action, predicts the occurrence of the next dynamic instants and assesses accordingly the start instant and the duration of its own action. In the remainder, the value of α (Equation 2) has been set to 0.4, while the size of the non-overlapping sliding time window is initialized to 2 s. The weight β in Equation (3) is kept to 0.5. Finally, we set ϵ, to define the width of the interval around the *targetTimestamp*, to the value 0.9.

## 6. Online Experimental Evaluation

In this section we discuss the results of the online experimental analysis we performed on the iCub, considering the task and the methodologies introduced before. We start with a qualitative assessment of the robot behavior on specific fragments of the interaction sessions, and later we summarize the overall performance on the entire sample set.

### 6.1. Qualitative Evaluation of Action Execution

**Figure 9:**
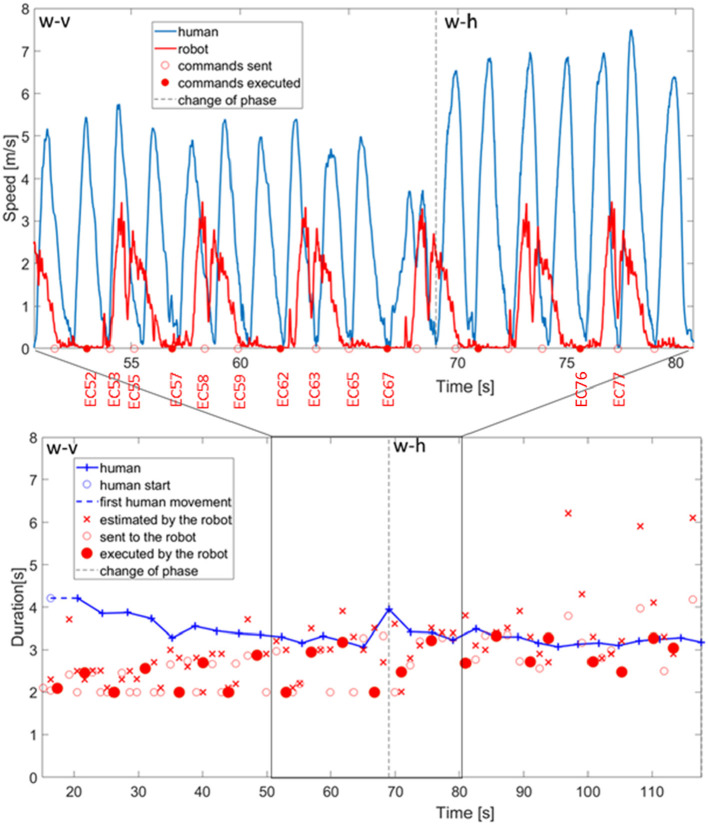
Above, bell shaped profile of human and robot actions with the commands of movement execution sent to the robot and the ones actually executed. Below, duration of human actions estimated by the robot, duration sent to the controller of the robot, and duration actually executed by the robot.

We start with a detailed description of how the robot coordinated with the human in the execution of the coordinated stamping task. In Figure 9, top panel, the data recorded from the motion capture for the coordination between the robot and a representative subject during phases w-v and phase w-h are reported. The detailed quantitative measures of the adaptation are reported later in this section. This figure is meant to provide a snapshot of the type of behaviors exhibited by the robot in the interaction. In this example, the robot infers the duration of human action in twelve reaching movement recorded from the first phase (w-v: wide and vertical movements). In the following, we will refer to the command planned at time t as EC <t>.

With the command of action execution planned by the robot at 52.0s (EC52—indicated with a full red circle in the x-axis of Figure 9, top panel), the robot starts the execution of a stamping action which comprises first 800 ms of no-movement, constituted by two bell shaped velocity profiles, that synchronize with the execution of a back and forth human stamping (two bell shaped velocity profiles, marked in blue). The action commands EC53, EC55 are planned in agreement with the human dynamic observation but never executed by the robot motor control system. In fact, the algorithm is designed to neglect action commands that occur when the previous motor action is still in execution. On the other hand, the command EC57 is executed and generates two movements that synchronize with the two human movements. The small movements of the robot after the motor command and before the consistent acceleration to the velocity maximum are small preparatory adjustments of the joint angles. The adjustment is also considered in the analysis of the duration of human and robot actions (see Figure 9, bottom panel). Both the timing of the action-start and action-stop events (and consequently the duration) are aligned with human actions and minima of both the human and robot movements coincide. EC58 and EC59 commands are ignored since the robot is executing previous motor commands. The EC62 triggers another motor action that ends after the E63, and E65 commands are generated.The execution of the successive action starts with EC67 but in that precise moment the human participant changes the direction and the speed of the movement to initiate phase w-h. The dashed vertical line indicates the change of the next phase and consequently the beginning of the first reaching movement in the phase w-h. The monitoring and coordination with the human individual adapts to the pace change and the robot executes an action (execution command EC76) that has its minimum aligned with the minimum of human action. However, it is clear that the duration can be further adjusted.

### 6.2. Robot Adaptation to Human Action Pace

The monitoring of human actions guides automatic adjustment of robot pace, as can be seen also from Figure 9, bottom panel. In this figure, the phase changes are indicated by vertical dashed lines and the duration of action executions for both the subject 5 (in blue color) and the robot (in red color) are reported. In particular, the cross marker represents the duration estimated by the robot, the circle marker represents the action command sent by the robot but not actually executed, whereas the full circle marker indicates the motor commands actually executed by the iCub.

The initial *a priori* estimate of human action duration is 2.0 s, and starting from this value, the duration estimated by the robot converges to the real value through the observations of the partner's movements. Considering the duration of the actions planned by the robot (both for those that are actually executed and the ones which are just planned), it can be seen that some them are of reduced duration. We limited the duration of robot's actions to a minimum of 2 s in order to avoid the execution of too fast actions, which could have been perceived as dangerous by the human partner. These correspond to anticipatory action commands that are programmed to synchronize the robot with a lost human action without stopping the robot execution. The advantage of such approach is that the robot action pace is never interrupted and flows smoothly across the entire collaboration.

Before the change from phase w-v and phase w-h, as we have seen in the graph for the specific case, there is a tendency to underestimate before *t* < 50 s. During the change from phase w-v and phase w-h the human participant increases the duration of action execution. The robot estimation becomes generally correct despite this small adjustment, but the system overestimates the duration at 96.95, 108.2, 116.4 s because of minima lost in the monitoring process. We considered correct response when the estimation error is <2 s (upper boundary for coordination). However the executed motor commands are only partially affected by this error thanks to the filtering component (as indicated in Equation 3) in the estimation of the human actions duration.

### 6.3. Robot Challenges in Online Scenarios

After having described in detail the behavior of the robot from the analysis of the interaction with one subject, we assessed the average performance of the system in the whole sample. Before reporting the results, it is worth identifying and provide a quantification of the challenges the robot needs to address to grant an adaptive behavior.

**Figure 10:**
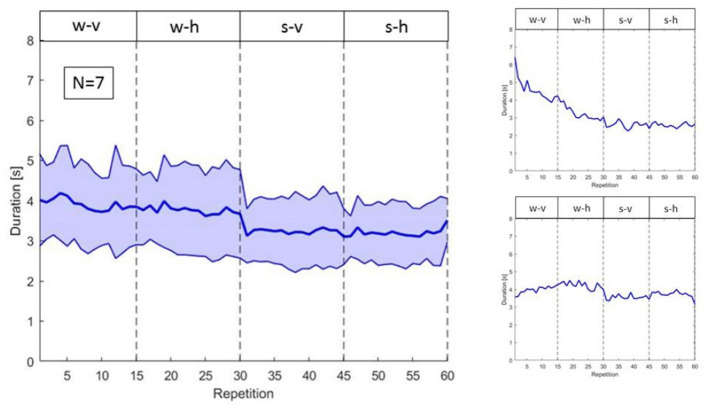
On the left, variability of the duration of human actions in the different phases. On the right, duration of actions of subject 2 (above) and subject 7 (below).

First, in this interactive scenario iCub has to address variability occurring both between and within subjects. In fact, although subjects were instructed to perform the same reaching actions in the same order, they started at different execution speeds and adjusted such execution differently over time, as shown in Figure 10. The reaching and porting movement, computed from the recordings of the motion capture system, are executed on average in ~4 s for the w-v and w-h phases and in 3.5 s for the s-v and s-h phases. More importantly the variability across subjects is relatively large (standard deviation for the w-v and w-h phase is approximately ±1 s and for the s-v and s-h ±1 s, see Figure 10, left panel).

Further, there is also variability within subjects, with phenomena like habituation leading to change in action duration over time. For instance, the two right panels in Figure 10, show how two participants, respectively subject 2 and subject 7, habituate differently to the task during the 60 repetitions.

Furthermore, the actions performed by humans are assessed by the robot through vision. This implies that a movement in three dimensions is projected on the image plane (i.e., point of view of the robot on the scene), thus producing different apparent motions as a function of the relative position of the robot camera and the human agent.

**Figure 11:**
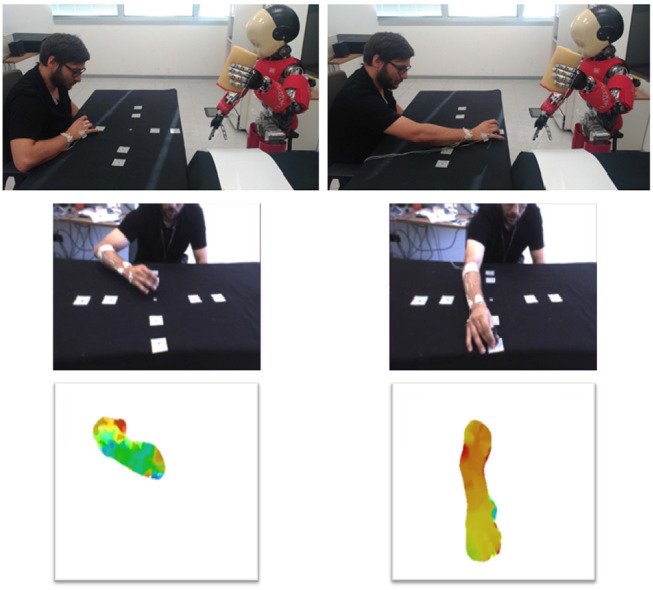
Start and end of the movement viewed by the Optotrak (above) and by the robot (middle). Below, the optical flow extracted by the robot.

In Figure 11, we can appreciate how the initial and final part of the reaching movement generate different optic flows due to different moving body parts (i.e., arm, forearm, and hand). Also the typology of the generated optic flow varies between the phases of orthogonal movement (phases h) and movements directed toward the robot (phases v). In the first, the optic flow direction is close to be orthogonal to the image plane and in the latter the optical flow expands in the image plane.

**Figure 12:**
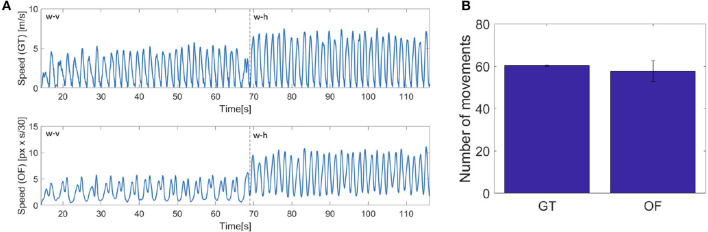
**(A)** On the left, speed computed from the Optotrak data (GT) and from the optical flow extracted by the robot camera (OF) for the 5th subject. **(B)** On the right, mean of the number of actions detected *a posteriori* from the two speed signals for all the subjects and all the phases.

**Figure 13:**
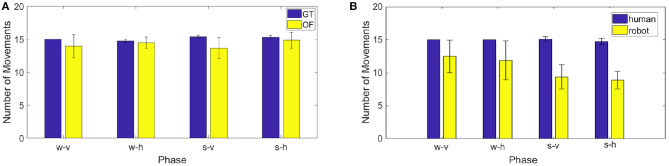
**(A)** On the left, number of actions detected *a posteriori* from the data recorded by the Optotrak (GT) and from the optical flow extracted by the robot camera (OF) in the different phases. **(B)** On the right, mean of the number of movements performed by the human and by the robot in the different phases for all the subjects.

The projection on the image plane generate velocity bells intrinsically different from those acquired with the motion tracking system, namely our ground truth (GT). As it can be seen in Figure 12, while the velocity extracted from the optic flow (OF) is quite similar to that measured with the Optotrak (GT) when human motion is orthogonal to the robot (h), when the human moves his hand back a forth toward iCub (v), the two velocity profiles differ, with unimodal hand velocity peaks being perceived by the robot as bimodal. This is due to the fact that while the end-effector reaches the target the rest of the arm adjusts (i.e., elbow movements) and the movement is detected in the optical flow. As a consequence the optical flow never completely reaches zero when the arm is fully involved in the movement. This has already an effect, although modest, in the process of minima detection performed on the ground truth (GT) and optic flow (OF) measures. The difference is reported in the figure as number of movements in average across the experimental trials extracted from the two series GT and OF. To check the similarity between the two estimates of velocity (GT and OF) we computed *a posteriori* for all subjects the number of local minima for each phase in the two variables (see Figure 13, left). The two estimates are not significantly different for phases w-h and s-h (as the data are not normally distributed, we performed a Wilcoxon signed-rank test on the difference: *p* = 0.81, *Z* = 9; *p* = 0.63, *Z* = 7, respectively) whereas a significant difference is present for phase w-v and s-v (Wilcoxon signed-rank test on the difference: *p* = 0.03, *Z* = 21; *p* = 0.03, *Z* = 21, respectively), suggesting a more important discrepancy between the velocity profiles extracted from the optic flow from ground truth when the motion is performed toward the robot.

### 6.4. Experimental Evaluation on the Whole Sample

Having considered the challenges to be tackled by the system, we first analyzed how the robot coordinated with the subjects by comparing number of movements it executed with the number of human movements in each phase. As shown in Figure 13 on the right, the robot performs less movements than the human in all phases, with the difference becoming larger in the phases involving shorter (and faster) human movements.

**Figure 14:**
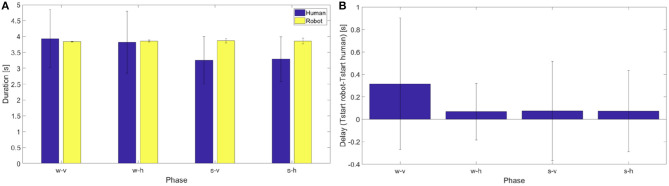
**(A)** On the left, average duration and standard deviation from the mean of human and robot actions in the different phases for all the subjects. **(B)** On the right, delay between human and robot movements.

A series of Wilcoxon signed-rank tests (as the data are not normally distributed) on the difference between the number of human actions and the corresponding number of robot actions shows a significant difference in the phases s-v and s-h (*p* = 0.02, *Z* = 28; *p* = 0.02, *Z* = 28, respectively) while it does not show a significant difference in the phases w-v and w-h (*p* = 0.06, *Z* = 20; *p* = 0.06, *Z* = 20, respectively). A Kruskall-Wallis test (as the data are not normally distributed) on the difference between the number of human actions and the number of robot actions with “phase” as factor, indicates that such discrepancy is not significantly different from one phase to another. In the following analyses we have then taken in consideration only those human actions for which the robot performed a corresponding coordinated action. To select them we considered the start instants of each robotic action and we individuated the human action which started more closely in time to it. For these subset of actions we compared action duration—between human and robot—and we estimated robot delay, as the average difference in time between each human dynamic instant and the closest action start. The average duration of the movement in the action execution between the robot and the participants (see Figure 14 left panel) are quite consistent. For all the phases the durations of the human and robot actions (which are normally distributed) were not significantly different [one sample *t*-tests on the differences: *t*_(6)_ = 0.22, *p* = 0.83 for phase w-v, *t*_(6)_ = −0.23, *p* = 0.83 for phase w-h, *t*_(6)_ = −2.28, *p* = 0.06 for phase s-v, *t*_(6)_ = −2.25, *p* = 0.07 for phase s-h].Since *p*-values are close to significance we cannot exclude that additional subjects might reveal a significant difference.

From the figure it is possible to note that for the duration in action execution the first two phases are different from the second two phases. In particular for the phases s-v and s-h the change in human movement duration reflects the fact that the distance of porting action is shorter. The monitoring process of the system detects a change in the human actions, but the action execution is only partially adjusted as demonstrated by the increase of the standard deviation of the last phases. However, a one-way RM anova on the difference between the duration of human actions and the duration of robot actions with “phase” as factor, fails to individuate a significant difference among the phases [*F*_(3, 24)_ = 1.23, *p* = 0.321].

Considering together these results, we conclude that the estimation of the human action duration is reasonable but not precisely accurate. This explains the reduced number of robot movements in the phase s-v and s-h with respect to human movements.

Last, in Figure 14, right panel the delays of action execution between each participant and the robot are reported for each phase. As the data are normally distributed, we performed a series of one sample *t*-tests that shows that for all the phases the delay was not significant [*t*_(6)_ = 1.08, *p* = 0.32 for phase w-v, *t*_(6)_ = 0.67, *p* = 0.53 for phase w-h, *t*_(6)_ = 0.20, *p* = 0.85 for phase s-v, *t*_(6)_ = 1.08, *p* = 0.32 for phase s-h]. It is worth noting though that this analysis was performed on the subset of human action that were closer in time to the actions actually instantiated by the robot. We then performed a one-way RM anova on the delay, and the results [*F*_(3, 24)_ = 0.3, *p* = 0.8237] say that no phase is significantly different from the others.

We conclude that despite the wide variability of the different subjects, the monitoring and coordination system of the robot can synchronize with the human partner, at least when his or her movement is of sufficient width and duration to be properly perceived online by the robot cameras. Some considerations about the huge variability between and within subjects indicate the rate at which estimated duration adjusts should be improved. In particular, the rate should be subject dependent and should accommodate individuals that vary considerably their pace of repetitive movements.

## 7. Discussion

With this study we investigated the use of a motion signature that relies on the detection of dynamic instants—points where the motion is subject to a change—and dynamic intervals—portions of the velocity profile of a movement delimited by a pair of dynamic instants—to the purpose of understanding actions in unconstrained collaborations, and achieving synchronization in collaborative HRI tasks characterized by a repetitive action. The method has been designed to work without *a priori* knowledge of the actor's posture or the trajectory of his/her action execution. In addition the solution is robust to change in the perspective from which the action is observed.

Overall, it is our opinion that, despite necessary fine tuning of the parameters, both action understanding and synchronization enable dependable collaboration between the robot and the human. The solution we propose is particularly well-suited for robotics, in that it is “agnostic” to *a priori* knowledge and sensors quality: it requires only a single RGB camera, as well as only a very short training dataset, providing a simple and versatile approach to these tasks. The use of dynamic instants to classify the action and to detect opportune coordination timing constitutes an advantageous solution, with the potential of promoting natural interaction and facilitating efficient mutual understanding between the two coordinated agents.

More precisely, in the sections 2, 3 we showed how the method, despite its simplicity, is able to capture similarities between actions in unconstrained interaction settings. This suggests that the motion signature incorporates very relevant motion properties that may be further exploited for more complex understanding tasks. A first straightforward extension concerns the level at which the descriptor is built. In this work, the motion signature incorporate information from the whole video and thus is a way of representing *repetitions of actions* rather than a single action instance. However, for favoring collaborations also in challenging scenarios with free and unconstrained possibilities of interaction, a more detailed representation is needed to associate semantic information with action segments. This is turn enables the capability of understanding *when* and *where* to intervene. For all these reasons, we are now working on the design of novel motion representations combining the information derived from dynamic instants and intervals at multiple temporal scales, to cope with single instance action recognition but accounting for the possible different durations in time of actions.

From section 4 on, we presented the implementation of the proposed method in the iCub framework, to detect and predict dynamic instants occurrences and consequently plan reaction with appropriate action executions. To validate the system in an interactive online context, we considered a proof-of-concept scenario, where the robot had to execute a stereotyped action in coordination with a human performing its own task. Our results provide strong evidence of the potential of the proposed approach, which proved also to be robust to drastic changes in the properties of the sensors adopted in the action observation (e.g., frame rate or camera resolution).

Our work can be extended in several different directions. Currently, conflicts in the interaction are handled so to guarantee a continuous action flow, favoring coordination and mutual adaptation. Future works will specifically consider situations in which the stability of the robot action duration is more crucial, so as to diversify the robot response depending on the context.

The fundamental aspect of our implementation is that the method assumes observation of repetitive actions and it promotes coordination only when parameters are fine-tuned for the specific individual. In the future we will consider an “observation phase” in which the robots monitors the human partner behaviors (including the computation of 3d optical flow), infers the class of actions and evaluates the stability of the timing, before deciding the parameters of the action coordination.

The extension to full body motion will be also object of investigation in future works. Given the strong assumption we are making when we collapse all the information derived from the optical flow in a single point, i.e., that there is just one main body part conveying relevant information about the observed movement, the method in the present form is going to fail in situations where such assumption is not valid, as in the case of full body motion. In such circumstance, a more refined pre-processing of the low-level features is needed, first identifying the body parts and then building a description for each of them, possibly combining their different contribution in a later stage.

## Declaration

The authors hereby declare that all the photos in the manuscript are by the authors and by their collaborators, and they all consent for the photos to be published in the manuscript.

## Ethics Statement

This study was carried out in accordance with the recommendations of the protocol IIT_INT_HRI—Ricerca sullo sviluppo dell'interazione sociale uomo-uomo e uomo-robot nel bambino e nell'adulto, with written informed consent from all subjects. All subjects gave written informed consent in accordance with the Declaration of Helsinki. The protocol was approved by the Comitato Etico Regionale della Liguria.

## Author Contributions

All authors listed have made a substantial, direct and intellectual contribution to the work, and approved it for publication.

### Conflict of Interest Statement

The authors declare that the research was conducted in the absence of any commercial or financial relationships that could be construed as a potential conflict of interest.

## Footnotes

^1^Notice in this work we use the terms *action* and *activity* interchangeably, although they refer in general to different concepts.

^2^https://github.com/nicolettanoceti/cookingdataset
